# Age-Appropriate Functions and Dysfunctions of the Neonatal Neutrophil

**DOI:** 10.3389/fped.2017.00023

**Published:** 2017-02-28

**Authors:** Shelley Melissa Lawrence, Ross Corriden, Victor Nizet

**Affiliations:** ^1^Pediatrics, Neonatal-Perinatal Medicine, UCSD, La Jolla, CA, USA; ^2^Division of Host-Microbe Systems and Therapeutics, UCSD, La Jolla, CA, USA; ^3^Pharmacology, UCSD, La Jolla, CA, USA; ^4^Skaggs School of Pharmacy and Pharmaceutical Sciences, UCSD, La Jolla, CA, USA

**Keywords:** neonates, neutrophils, granulopoiesis, innate immunity, phagocytosis, chemotaxis, neutrophil extracellular traps, transmigration

## Abstract

Neonatal and adult neutrophils are distinctly different from one another due to well-defined and documented deficiencies in neonatal cells, including impaired functions, reduced concentrations of microbicidal proteins and enzymes necessary for pathogen destruction, and variances in cell surface receptors. Neutrophil maturation is clearly demonstrated throughout pregnancy from the earliest hematopoietic precursors in the yolk sac to the well-developed myeloid progenitor cells in the bone marrow around the seventh month of gestation. Notable deficiencies of neonatal neutrophils are generally correlated with gestational age and clinical condition, so that the least functional neutrophils are found in the youngest, sickest neonates. Interruption of normal gestation secondary to preterm birth exposes these shortcomings and places the neonate at an exceptionally high rate of infection and sepsis-related mortality. Because the fetus develops in a sterile environment, neonatal adaptive immune responses are deficient from lack of antigen exposure *in utero*. Newborns must therefore rely on innate immunity to protect against early infection. Neutrophils are a vital component of innate immunity since they are the first cells to respond to and defend against bacterial, viral, and fungal infections. However, notable phenotypic and functional disparities exist between neonatal and adult cells. Below is review of neutrophil ontogeny, as well as a discussion regarding known differences between preterm and term neonatal and adult neutrophils with respect to cell membrane receptors and functions. Our analysis will also explain how these variations decrease with postnatal age.

## Introduction

The creation of life through human pregnancy is an astonishing achievement of nature, by which the allogeneic fetus is protected from maternal rejection through placental separation of fetal and maternal vascular systems as well as immunosuppression resulting from high levels of maternal progesterone and placental production of glucocorticoids (1). Intriguingly, this protection ensues even if the fetus is genetically diverse from its carrier, as observed with surrogate pregnancies, indicating a similarly tolerant fetus. Although suppressed, maternal immunity provides primary protection to the developing fetus against *in utero* infections and helps safeguard the newborn during the first year of life. This protection is achieved not only through placental transport of maternal immunoglobulins during the last trimester of pregnancy (2) but also by the newborn’s consumption of breast milk, rich in antimicrobial proteins, immunoglobulins, and beneficial oligosaccharides (3). If maternal defenses are breeched by pathogens, resulting in chorioamnionitis or neonatal infection, a detrimental inflammatory cascade may be initiated in the neonate with the potential for devastating long-term neurodevelopmental sequelae (4, 5) and/or perturbations in the normal development of the immune system (6).

Because the fetus develops in a sterile milieu, neonatal adaptive immune responses are naïve from lack of antigen exposure *in utero*. Newborns must, therefore, rely on innate immunity to protect against early infection (7–9). Neutrophils are a vital component of innate immunity because they are the first circulating immune cells to respond to and defend against bacterial, viral, and fungal infections. However, notable phenotypic and functional disparities exist between neonatal and adult cells. The severity of these impairments is inversely related to gestational age (GA), revealing progressive neutrophil maturation throughout pregnancy, from the earliest hematopoietic precursors in the yolk sac to the well-developed myeloid progenitor cells in the bone marrow around the seventh month of pregnancy. These deficiencies place our most vulnerable patients at risk for infection and sepsis-related mortality.

Environmental factors also directly impact neutrophil phenotype and function, and differ considerably between the maturing fetus and adult. The intrauterine environment is exceedingly hypoxic with oxygen concentrations measured in the range of 1–5%, compared to 21% in the Earth’s atmosphere. This low oxygen content necessitates cellular suppression mechanisms to counteract hypoxia-inducible factor 1α (HIF-1α)-mediated pro-inflammatory gene expression. Similarly, immune tolerance is vital during and after parturition, when the neonate is newly exposed to trillions of microorganisms that will become important components of its healthy microbiome.

For more than 50 years, scientists have been striving to understand the intrinsic mechanisms underpinning the normal transition of the suppressed *in utero* neutrophil into the fully functional postpartum cell capable of combating pathogenic organisms. This quest is even more urgent for extremely premature neonates, who are born at the limits of viability, and join the world before the immune developmental program is properly executed. As a consequence, these vulnerable neonates experience a profound compromise of both innate and adaptive immune responses. In this review, we explore differences between neonatal and adult neutrophils, describe neutrophil maturation throughout pregnancy, and highlight therapies trialed in neonates to enhance neutrophil function.

## Development

### Hematopoiesis

Fetal hematopoiesis, or the creation of all blood cells, is an evolutionarily conserved process that originates in the extra-embryonic yolk sac around the third week of embryogenesis and gives rise to a transient population of primeval erythroid cells, macrophages, and megakaryocytes (10, 11). Around the seventh to eighth week of gestation, genuine hematopoietic stem cells (HSCs) are derived from specialized intra-embryonic endothelial cells located in the ventral wall of the descending aorta (12–14). These self-renewing primitive HSCs, with increased proliferation potential (15), will seed the liver, thymus, and spleen, where hematopoiesis will continue until the seventh month of gestation (10, 16). After this time, hematopoiesis will transition to the bone marrow, such that by the end of term gestation, the bone marrow becomes the primary source of red cells, white cells, and platelets (17, 18).

Neutrophils first appear in the human clavicular marrow at 10–11 weeks post conception (19). By the end of the first trimester, neutrophil precursors are detected in the peripheral blood, while mature cells appear by 14–16 weeks of fetal development (20, 21). HSCs that generate neutrophils are situated in specialized niches in the trabecular regions of long bones near the endosteum, or the interface between the bone and bone marrow, in proximity to osteoblasts (22–24). To exit the bone marrow, neutrophils must traverse the bone marrow endothelium through tight-fitting pores by a process known as transcellular migration, whereby the cells pass through the cell bodies of the endothelium rather than through cell junctions (25, 26).

Neutrophils reside in three different groups, or pools, known as the proliferative, circulating, and marginating pools, with numbers in each influenced by the maturational development of the cell and the individual’s state of health. A delicate balance between neutrophil maturation, bone marrow storage and release, intravascular margination, and migration into peripheral tissues is closely regulated by conventional dendritic cells through the controlled production of granulocyte colony-stimulating factor (G-CSF), CXCL1, CCL2, and CXCL10 (27).

### Proliferative Bone Marrow Pool

The proliferative pool comprised mitotic neutrophil precursors, including myeloblasts, promyelocytes, and myelocytes, which maintain their ability to multiply in order to replenish neutrophil numbers (28, 29). In human adults, the proliferative pool is estimated to contain between 4 and 5 × 10^9^ cells/kg bodyweight (30, 31). In term neonates, however, this pool is greatly diminished at only 10% of adult values, with more than two-thirds of their cells residing in an active cell cycle, resulting in substantial cell turnover (20, 32). The absolute neutrophil cell mass per gram body weight in term neonates is also considerably less, calculated to be only one-fourth that of adult levels (33), while preterm infants <32 weeks of gestation exhibit even lower values (~20% adult numbers) (34) (Table 1).

**Table 1:** **Variances between neonatal and adult neutrophils**.

Variable	Preterm	Term	Matures	Comment
Neutrophil cell mass (per gram BW)	↓↓	↓	Yes	Adult levels achieved by 4 weeks of age (20, 34)
Storage pool	↓↓	↓	Yes	Reduced storage pools lead to increased risks for neutropenia if infection occurs postnatally (20, 37)
Number in circulation	↑/↑	↑	Yes	Increases noted for all gestational age (GA) infants in the first 24 h after birth. Quantities return to adult levels by 72 h of life. The highest levels are found in neonates <28 weeks of GA (64)
Number of IG in circulation	↑	↑	Unknown	Neutrophil composition approximates that of adults by 72 h of life (65, 90)
Granule protein levels				
BPI	↓↓	↓	Yes	(68)
Lactoferrin	↓↓	↓	Yes	(75)
Chemotaxis	↓	↓	No	Factors include reduced mobilization of intracellular calcium (88) and anomalies in cytoskeletal organization (89)
Rolling and firm adhesion				
L-selectin levels	↓↓	↓	Yes	(8, 93)
L-selectin shedding	↓↓	↓	Yes	(8, 93)
CR3	↓↓	↓	Yes	(38, 93)
Transmigration	↓↓	↓	Yes	Decreased secondarily to reduced levels of CR3 and diminished release of chemokines and cytokines from tissue neutrophils and macrophages (38, 93)
Neutrophil extracellular trap (NET) production	↓↓	↓	Yes	Neonatal neutrophils only produce NETs in a ROS-independent manner (152, 153, 157)

*↑, increased; ↓, decreased; IG, immature granulocyte; BPI, bactericidal permeability-increasing protein*.

Neutrophils are activated from the early phases of bloodstream or deep tissue infection, causing their numbers in circulation to rapidly rise (35). Hence, bone marrow reserves of mature neutrophils are rapidly depleted due to limited stores, necessitating the release of immature granulocytes (IGs), known as a “left shift,” which is often used to assess a person’s probability of serious infection or sepsis (36). Because newborns, particularly very low birth weight premature neonates, have an exceptionally limited ability to recruit or generate significant neutrophil numbers, they are more likely to develop neutropenia when confronted by a pathogenic challenge, thereby increasing sepsis-associated morbidity and mortality (16, 20, 37). By contrast, adults maintain a large number of quiescent neutrophil progenitors that can be rapidly recruited into the cell cycle during times of sepsis (16, 20, 37, 38), coupled to a sizable bone marrow reserve of near-mature and mature neutrophils that can be quickly mobilized in early inflammatory responses (20 times that found in circulation) (30). Ultimately, neutrophil numbers in term and preterm infants will rise over the first few weeks of life to achieve adult values by 4 weeks of age (34).

### Diminished Neutrophil Production and Neonatal Neutropenia

Neonates who are small for gestational age (SGA) at birth, or have a birthweight <10th percentile, also have higher rates of neutropenia (absolute neutrophil count of <1,000/mL) compared to non-SGA infants, with an incidence of 6 vs. 1%, respectively. SGA neutropenia usually persists for the first week of life and is associated with thrombocytopenia in more than 60% of neonates (39). Previous conclusions of a direct correlation between neutropenia and preeclampsia (40), or related placental deficiency, have since been disproven with regression models demonstrating no higher incidence of low neutrophil counts over and above that calculated for SGA alone (39, 41). SGA neutropenia most likely results from *in utero* growth restriction rather than high maternal blood pressure because the severity of neutropenia is directly correlated with the number of circulating nucleated red blood cells (39). Diminished neutrophil production, rather than accelerated neutrophil destruction or excessive margination, is considered the primary mechanism underlying this phenomenon, as a normal immature to total (I:T) neutrophil ratio is maintained (39). Additionally, findings of decreased neutrophil production from pluripotent hematopoietic progenitors, reduced concentrations of granulocyte-macrophage progenitors, diminished bone marrow neutrophil proliferative and storage pools, and absence of evidence for excessive margination have been found in experimental models (40). Impaired neutrophil production is therefore thought to result from (1) downregulation of neutrophil growth or transcription factors due to high concentrations of erythropoietin (42), (2) inadequate G-CSF production, or (3) a placental inhibitor of neutrophil production that has yet to be identified (39). Notably, neutropenic SGA infants have an increased probability of being diagnosed with late-onset sepsis, as well as a fourfold increased risk of developing necrotizing enterocolitis for reasons that remain unclear (39).

Intriguingly, extremely low birth weight (ELBW) infants, or those born less than 1,000 g at birth, experience the highest frequency of neutropenia of any other neonatal group without an identified cause (43). Unlike older gestational aged neonates, neutropenia in ELBW infants is usually not associated with sepsis (44). Therefore, ELBW infants with and without neutropenia experience similar mortality rates in the NICU (43).

Trials investigating the clinical use of recombinant G-CSF and granulocyte-macrophage colony-stimulating factor (GM-CSF) to increase neutrophil numbers in preterm infants have yielded disappointing results, granted that sample sizes have been small. Although both drugs increased overall neutrophil numbers, no differences in mortality were observed by day 14 from the start of therapy in preterm infants with suspected or proven systemic infection who received concurrent antibiotic therapy (45, 46). The PROGRAMS trial, which specifically studied the prophylactic use of GM-CSF in SGA preterm infants in the first 5 days of life, also found no benefit in sepsis-free survival to day 14 from trial entry compared to the control group (47). In this study, GM-CSF was preferentially chosen because of its ability to illicit a T_H_1 immune response, prime neutrophils and monocytes to enhance bactericidal activity, and stimulate proliferation of neutrophil progenitors. A subgroup analysis by the Cochrane Group, however, identified 97 preterm infants from three studies, who suffered from both neutropenia and systemic infection at time of enrollment and received either drug. Remarkably, this defined group experienced a significant reduction in mortality by day 14 [RR 0.34 (95% CI 0.12–0.92); NNT 6 (95% CI 3–33)] (46), signifying that further appropriately powered studies should be undertaken to determine efficacy in this specific patient population (48).

### Granulopoiesis

**Figure 1:**
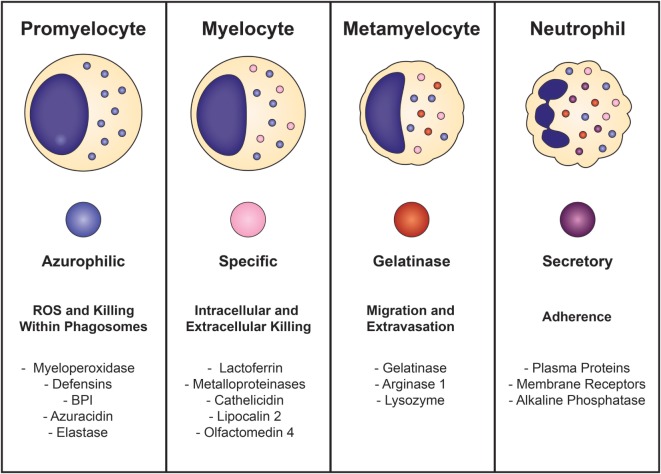
**Neutrophil granulopoiesis**. Neutrophil maturation is defined by the sequential formation of three different granules and secretory vesicles, as well as nuclear segmentation. Granulopoiesis begins with the development of azurophilic granules in myeloblasts/promyelocytes and concludes after creation of secretory vesicles in mature, segmented cells. Azurophilic granules, which contain acidic hydrolases and microbicidal proteins, fuse with phagosomes to form highly toxic enclosures for oxidative reactions, necessary for pathogen destruction. Specific granules participate in both extracellular and intracellular microbial killing and are rich in antibiotic substances. Gelatinase granules are mobilized when the neutrophil establishes rolling contact with inflamed endothelium and contain matrix-degrading enzymes (gelatinase) and membrane receptors. Secretory vesicles are the first to be mobilized after minimal neutrophil stimulation and contain membrane-associated receptors that are key for chemotactic-directed migration and the establishment of firm contact with activated vascular endothelium.

Neutrophil development is defined by the formation of granules within the maturing cell, known as granulopoiesis. This process begins between the myelocyte and promyelocyte stages of development and proceeds over the subsequent 4–6 days to produce mature, segmented neutrophils (Figure 1) (49–51). Formation of neutrophil granules occurs in a continuum by a process known as “*targeting by timing*,” whereby granule proteins are sequentially packaged as they are produced, so that azurophilic granules are synthesized in promyelocytes, specific granule proteins in myelocytes, and gelatinase granule proteins in metamyelocytes and band cells, after which granule formation concludes and secretory vesicles form (50, 52–54). Recent findings reveal that direct sorting of granule components is also imperative for proper neutrophil granule formation. This is exemplified by the proteoglycan serglycin, which is essential for the shuttling and packaging of α-defensin and elastase into azurophilic granules (55–57). Additionally, the discovery of adaptor protein complexes and the monomeric Golgi-localized γ-adaptin ear homology ARF (GGA)-binding protein have shown that together, these substances recognize, organize, and traffic granule proteins from the *trans*-Golgi network to their respective granule compartments based on complex co- and posttranslational processing similar to that of lysosomal sorting of other cell types (58, 59).

Neutrophil granules are exocytosed in the reverse order of their formation in a hierarchical fashion based on the function of their contents and the magnitude of the stimulus (50, 51, 60). Thus, secretory vesicles are the first to be extruded after minimal cellular stimulation or activation. Although not considered a true neutrophil granule, secretory vesicles are mobilized in the earliest stages of neutrophil-mediated inflammatory responses and are vital reservoirs of membrane-associated receptors that allow the neutrophil to establish firm contact with activated vascular endothelium and transmigrate into inflamed tissues. Gelatinase granules follow and contain matrix-degrading enzymes (gelatinases) and membrane receptors that are important for extravasation into inflamed tissues during early inflammatory processes. Specific granules are mobilized next and release their antimicrobial contents extracellularly or discharge their substances into phagosomes. Phagosomes provide lethal enclosures for the intracellular killing of phagocytosed microorganisms through the creation of confined spaces that allow for exposure to high concentrations of microbicidal proteins and hydrolytic enzymes. Azurophilic granules are the last to be mobilized and are unique among the neutrophil granules because they can only degranulate their contents into phagosomes after activation by very powerful stimuli. Because azurophilic granules contain acidic hydrolases and microbicidal proteins that can be harmful to surrounding tissues if released extracellularly, close regulation is necessary. Recently identified variations in soluble *N-*ethylmaleimide-sensitive factor attachment protein receptor (SNARE) complexes explain these differences: whereas all neutrophil granules contain syntaxin 4 and SNARE complexes within their membranes, specific and gelatinase granules have SNARE complexes with high concentrations of VAMP-1, VAMP-2, and 23-kDa synaptosome-associated protein 23, while azurophilic granules have increased levels of VAMP-1 and VAMP-7 (61, 62).

### Circulating and Marginating Neutrophil Pools

More mature neutrophils that reside outside of the proliferative pool (metamyelocytes, bands, and segmented cells) are found in equilibrium between the free flowing circulating pool and marginating pool (63). Remarkable fluctuations of the circulating pool occur in nearly all neonates after birth due to a surge in neutrophil numbers in the first 6–24 h of life to levels never again encountered in one’s lifetime while healthy (64). This rise occurs earlier in neonates ≥28 weeks of GA with peak levels of 25–28,000 cells/μL noted around 6–12 h of life, while in those <28 weeks of GA experience a more gradual but dramatic rise, with maximum numbers of up to 40,000 cells/μL being achieved around 24 h of life (64). Irrespective of GA at birth, all newborns will subsequently undergo a gradual decline in neutrophil numbers over the next 72 h, with neutrophil composition and counts closely approximating that of adults by the third day of life (64).

Differences in neutrophil composition also exist, as term neonates have an increased number of IGs (promyelocytes, myelocytes, and metamyelocytes) when compared to adults (12 vs. 5%, respectively) (65). The higher quantity of IGs, which are deficient in vital early pro-inflammatory proteins and receptors due to incomplete or absent development of gelatinase granules or secretory vesicles, may increase a neonate’s risk of infection after birth while simultaneously guarding against inappropriate inflammatory responses during the creation of its microbiome. Because neonates have a limited proliferative pool, neutrophils involved in this early surge are theorized to accrue from the marginating pool in response to birth-related stress hormones produced in the neonate, although the exact source and mechanism involved remain unknown.

## Functional Differences of Neonatal Neutrophils

### Microbicidal Proteins and Activity

Degranulation capabilities are similar between term neonatal and adult neutrophils, while those from preterm infants have considerable impairments in the release of bactericidal/permeability-increasing protein (BPI), elastase, and lactoferrin when compared to either term neonatal or adult cells (66, 67). Additionally, neutrophils from term healthy newborns and adults contain equal concentrations of the azurophilic granule proteins myeloperoxidase and defensin, while BPI is decreased threefold in unstimulated term neonatal neutrophils compared to adult controls (68). Interestingly, term infants with early-onset sepsis (EOS) experience a rise in plasma levels of BPI comparable to those of older children with sepsis syndrome (69) and adults with bacteremia (70) or pneumonia (67, 71). In laboratory studies using the stimulus phorbol myristate acetate (PMA), Nupponen and colleagues demonstrated that term and adult neutrophils generated similar concentrations of BPI, while its production remained significantly diminished in preterm neonatal cells. This finding suggests that BPI mobilization within the neutrophil exhibits an age-dependent maturational effect (67). BPI has a high affinity for the lipid A portion of lipopolysaccharide (LPS; the endotoxin of Gram-negative bacteria), thereby neutralizing its pro-inflammatory properties (68, 72). BPI also enhances phagocytosis of Gram-negative bacterium by acting as an opsonin (68, 73). These factors may explain why preterm infants deficient in BPI mobilization are more likely to become septic with Gram-negative bacteria, such as *Escherichia coli*, the leading cause of EOS in preterm infants. Additionally, the specific granule protein lactoferrin has direct bacteriostatic and bactericidal activities against viruses, Gram-positive bacteria, Gram-negative bacillis, and fungi (74). Lactoferrin measured in term neonatal neutrophils was half of adult concentrations, while preterm cells had even lower quantities (75).

Galectin-3, a S-type lectin receptor, is a non-traditional neutrophil membrane receptor with pro-inflammatory autocrine/paracrine effects on neutrophil phagocytosis, particularly of *Candida* species (76, 77). This receptor recognizes and binds to β-(1-2) oligomannan, thereby allowing the cell to distinguish between pathogenic and non-pathogenic fungi (76). Following its release extracellularly by activated or damaged neutrophils, galectin-3 binds to the neutrophil cell membrane. This binding results in the co-ligation of CD66a and CD66b, which leads to receptor clustering, integrin-mediated adhesion, and enhanced phagocytic capabilities (77). Galectin-3 has also been shown to increase reactive oxygen species (ROS) production, enhance neutrophil degranulation, and inhibit apoptosis (77–79). Although contrasting data exist regarding serum and plasma levels in term neonates and adults, serum levels appear to be lower in preterm compared with term infants and rises throughout gestation (76, 80). Galectin-3 levels have also been demonstrated to be higher in neonates delivered vaginally compared to cesarean without labor, which may prime labor exposed neutrophils and render them more responsive to challenges with Gram-negative bacteria (79) or fungi (76, 80).

### Chemotaxis and Migration

Neutrophils comprise the majority (60%) of leukocytes in humans (51) and are the “police force” of the immune system because they are the first immune cells to respond to and combat invading pathogens. During the earliest stages of infection or inflammation, chemoattractants derived from either the host (e.g., chemokines, cytokines, leukotrienes) and/or pathogen (e.g., LPS, fMLP) are released into the bloodstream, causing stimulation and activation of quiescent neutrophils. These agents also create a biochemical gradient that is sensed by specialized G protein-coupled receptors and induce intracellular signaling cascades that result in cell polarization, cytoskeletal rearrangement, and adhesion molecule clustering. These changes are necessary to enable the activated neutrophil to hone in and migrate toward the site of infection in the process known as chemotaxis (8, 81).

Neonatal neutrophils have similar chemotactic abilities, irrespective of GA (63, 82), but demonstrate reduced responsiveness when compared to adult cells (83–87). Though the number and affinity of cell surface receptors are comparable, deficiencies in neonates are attributed to reduced mobilization of intracellular calcium that result in aberrations in chemoattractant-induced signaling (88), as well as anomalies in microfilamentous cytoskeletal organization from delayed F-actin induction (89). IGs are also inept at chemotaxis and are found in higher numbers in neonates after birth as compared to adults (90, 91). In general, though, neutrophils from term infants achieved similar chemotactic abilities to adult cells by around 4 weeks of age. By contrast, deficiencies persisted in nearly half of preterm infants at 42 weeks of GA for reasons that remain unclear (84, 85), indicating that birth and extrauterine environmental factors do not fully correct the developmental program of maturation.

**Figure 2:**
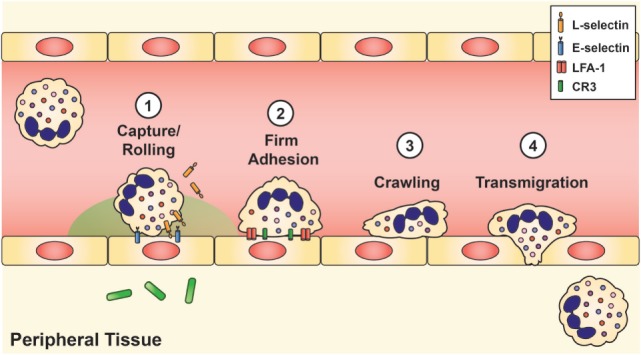
**Neutrophil recruitment and tissue extravasation**. Chemoattractants derived from the host and/or invading pathogens activate quiescent neutrophils and provide a chemical gradient for stimulated neutrophils to hone onto and migrate toward the site of infection. Once at the inflamed site, initial contact between the neutrophil and vascular endothelium occurs during rolling and capture, which is facilitated by L-selectin on the neutrophil and E-selectin on the inflamed endothelium. This initial contact causes shedding of L-selectin and triggers the induction of lymphocyte function-associated antigen-1 and CR3, which establishes firm adhesion. Neutrophils then exit the vasculature by paracellular migration at the endothelial borders (70–90%) or *via* transcellular passage (not shown).

Once the stimulated neutrophils arrive at the site of infection, extravasation into the tissue requires four mechanisms: (1) capture and rolling, (2) firm adhesion, (3) crawling, and (4) diapedesis or transmigration (Figure 2). Initial contact between the neutrophil and vascular endothelium occurs during rolling and capture, which depends upon the interaction of L-selectin on the neutrophil and P- or E-selectin on the endothelium. Once contact is established, chemokines located on the inflamed endothelium bind to specific chemokine receptors on the neutrophil cell surface, thereby triggering a conformational change of the neutrophil, shedding of L-selectin (92), and induction of β_2_ integrin expression, including lymphocyte function-associated antigen 1 (LFA-1) and CR3 (CD11b/CD18, α_M_β_2_, MAC-1), by inside-out signaling (93). L-selectin is then shed from the neutrophil, and the β_2_ integrins establish firm adhesion with the endothelium by binding to members of the immunoglobulin superfamily of adhesion molecules, such as intercellular adhesion molecule 1 and 2 (ICAM-1 and ICAM-2), vascular cell adhesion molecule 1 (VCAM-1), and receptor for advanced glycation endproducts (8, 50, 60).

Neonatal neutrophils exhibit marked impairments in slow rolling and adhesion. One reason is reduced expression and shedding of L-selectin (94). This cell membrane receptor first appears on neonatal neutrophils around 21 weeks of fetal development (38, 95), and its concentration on the neutrophil cell surface increases in an age-dependent manner (38, 93). Although L-selectin shedding improves with fetal maturation, overall quantity and ease of release remains far below that of adult levels at term gestation, with substantial deficits noted in neonates <30 weeks of GA (8, 38, 93, 96).

Another reason for deficient slow rolling and adhesion in neonates is diminished upregulation of CR3 following chemotactic stimulation. Intriguingly, levels of CR3 in mid-gestational aged neonates are similar to those of patients with leukocyte adhesion deficiency type 1 syndrome (38, 93). When cytosolic and cell membrane measurements of CR3 stores were analyzed, investigators found that neonates had only 10% of adult levels at 27 weeks GA, increasing to 48% at 36 weeks GA, and then to 57 ± 4% at term (38). Of note, adult levels of CR3 were not attained by neonatal neutrophils until 11 months of age (38). Conversely, the appearance of LFA-1 in the neutrophil cell membrane is not dependent on maturation, with equivalent abundance in adults and neonates, regardless of GA (8, 38, 97) (Table 1).

The ability of the neonatal vascular endothelium to upregulate its expression of adhesion molecules following exposure to LPS is also greatly reduced in an age-dependent manner (93, 98). Neonatal neutrophils also have impairments of transmigration through the vascular endothelium due to decreased quantities of CR3 and diminished release of chemokines and cytokines from tissue neutrophils and macrophages (47, 99). Thus, only about half as many term neonatal neutrophils transmigrate through the vascular endothelium in response to infection or inflammation when compared to adult cells (38).

Infant health has also been shown to affect chemotaxis. Remarkably, neonates who are ventilated for severe respiratory distress syndrome (but otherwise healthy) (84) and those with superficial infections (100) had neutrophils that exhibit enhanced chemotactic abilities when compared to those of stable preterm and term infants (84). Conversely, septic infants with Gram-negative bacteremia had poorly chemotactic neutrophils (82, 100). Likewise, intrapartum exposure to magnesium sulfate reduced neonatal neutrophil chemotactic abilities with impairment directly correlated with maternal serum magnesium levels (101), and insufficiencies related to antenatal betamethasone exposure were attributed to reductions in endothelial expression of adhesion molecules, including E-selectin, ICAM-1, and VCAM-1 (102).

### Phagocytosis

Neutrophils generally exit the vasculature by paracellular migration at the endothelial borders (70–90%), but a few will travel through the endothelial cell *via* a transcellular passage (103). Once within the tissue, oxygen deprivation created by the pathological conditions of the infection drives HIF-dependent activation of prosurvival pathways in the neutrophil (104, 105) to enhance its bactericidal activity (106). Neutrophils then continue to migrate along the chemotactic gradient toward the site of microbial invasion. When encountered, neutrophils engulf the pathogens using receptors for complement and the Fc domain of immunoglobulins, including CR1, CR3, FcγRII, and FcγIII (38). While the number of CR1 (107) and both Fcγ receptors (108) are the same between term neonates and adults, CR3 receptors are decreased as previously discussed (38, 109). Conversely, preterm infants at birth express 80–88% of adult levels of FcγRII but only 50% of FcγIII (109). FcγIII levels, however, rise rapidly postnatally to reach adult levels by 2 weeks of life. Together, these four receptors mediate binding, ingestion, and killing of bacteria (38) (Table 2).

**Table 2:** **Similarities between neonatal and adult neutrophils**.

Variable	Preterm	Term	Matures	Comment
Degranulation capabilities	↓	N	Yes	Only known for BPI, elastase, and lactoferrin (66, 67)
Granule protein levels				
Myeloperoxidase	N	N	No	(68)
Defensin		N	Unknown	(68)
Rolling and firm adhesion				
Lymphocyte function-associated antigen-1 levels	N	N	No	(8, 38, 97)
Phagocytosis	↓	N	Yes	Reduced in neonates with sepsis or non-infective clinical stress for some organisms (116)
CR1		N	Yes	(107)
FcγRII	↓	N	Yes	(108, 109)
FcγIII	↓	N	Yes	Adult levels in preterm infants reached by 2 weeks of age (108)
Respiratory burst	N/↑	N/↑	No	Decreased in stressed neonates or those with perinatal distress (134)
Chemiluminescence	N/↓	N/↑	No	Reduced in critically ill neonates and those challenged with large bacterial loads (139, 142)

*N, normal; ↑, increased; ↓, decreased*.

Neonatal neutrophils from term, healthy infants opsonize and ingest both Gram-negative (91, 110) and Gram-positive bacteria with equivalent efficiency to adult cells (111). By contrast, phagocytosis was less proficient in preterm infants <33 weeks of GA, who experienced both slower uptake and ingestion of bacteria compared to term neonates and adults. Curiously, these impairments persisted at 1–2 months of age, despite no apparent maturational defect (112). These deficits are believed to result from low circulating levels of opsonization factors, particularly maternal immunoglobulins that are actively transported across the placenta in the last trimester of pregnancy. Supportive evidence comes from the observation that administration of intravenous immunoglobulins (IVIG) normalizes phagocytosis capacity in preterm infants <32 weeks of GA (113). The use of IVIG in the treatment of neonatal sepsis, however, failed to reduce either (1) mortality during the hospital stay or (2) death or major disability at 2 years of age in infants with suspected or proven sepsis in a large cohort of neonates (114, 115). In the face of infective or non-infective clinical stress, the ability of neonatal neutrophils to phagocytose Gram-positive bacteria remained intact, but was impaired for Gram-negative bacteria (116).

Conversely, phagocytosis of *Candida* not only depends upon the size and form of the pathogen (i.e., hyphae or yeast) but also upon the species (76, 117). Destin and coworkers concluded that neutrophils from preterm and term infants as well as adult controls all failed to phagocytose unopsonized *Candida albicans* yeast, yet were similarly capable of phagocytosing unopsonized *Candida parapsilosis* (77, 117). Likewise, oxidative burst was equally robust when challenged with *C. albicans* hyphae in all groups, but non-existent against *C. parapsilosis* and attenuated against *C. albicans* yeast forms (117). Others have also demonstrated reduced phagocytosis and killing of *C. albicans* by preterm neonatal neutrophils due to deficiencies of opsonization factors (66, 118–120). Likewise, galectin-3, mentioned earlier in this review, is critical for the neutrophil’s ability to recognize, engulf, and kill pathogenic *Candida* species. This finding is most likely due to the ability of galectin-3 to co-ligate neutrophil cell membrane receptors CD66a and CD66b, resulting in cell receptor clustering and integrin-mediated adhesion (77). Galectin-3 also primes/activates the neutrophil, thereby enhancing ROS production, prolonging cell survival, and increasing degranulation of microbicidal proteins and substances (77).

### Phagocytosis-Associated Respiratory Burst and Chemiluminescence (CL)

Microorganisms, engulfed by neutrophils, are trapped within phagosomes that fuse with azurophilic and specific granules to form phagolysosomes, which are small, confined spaces designed for toxic, oxidative reactions vital in destroying pathogens while protecting the host tissue against harmful metabolites (121, 122). The formation of phagolysosomes is associated with an increase in hexose monophosphate shunt metabolism of glucose and, in turn, a proportional rise in molecular oxygen consumption that is known as the respiratory burst (121). NADPH oxidase, localized on the membrane of the phagolysosome, is also activated by phagocytosis and is essential in driving the respiratory burst *via* the reduction of oxygen (O_2_) to yield hydroxydioxylic acid (HO_2_) and hydrogen peroxide (H_2_O_2_) (121, 123). Inactivating mutations in NADPH oxidase result in chronic granulomatous disease (CGD), which is characterized by recurrent bacterial and fungal infections, as well as granulomas that result from the neutrophils’ inability to completely kill and eliminate pathogens (124). HO_2_ and H_2_O_2_ are weakly bactericidal (125) and lead to considerable acidification of the phagolysosome (95, 121). Myeloperoxidase, also released from azurophilic granules, catalyzes oxidation reactions between H_2_O_2_ and chloride (Cl^−^) to form hypochlorous acid (HOCl) (126), hydroxyl radicals (⋅OH), and chloramines, all of which are potent oxidants (127) that further contribute to the microbicidal capabilities of neutrophils (125). The resulting reactions between microorganisms and oxygenation radicals produce electronic excited products that cause light emission in the visible spectrum, known as CL (128).

The generation of ${\text{O}}_{2}^{-}$ can be detected using the nitroblue tetrazolium (NBT) test (129), which remains negative (clear) in patients with CGD, but produces a positive (or blue) reaction in neutrophils from healthy term newborns. Neonatal neutrophils induce an intensely positive reaction that corresponds to enhanced oxygen consumption in the initial phase of the respiratory burst and normal or elevated production of H_2_O_2_ (130). The NBT is also more intense from cord blood neutrophils exposed to labor than those without, suggesting that parturition primed these cells for increased activity (131, 132). Quantitative differences in the kinetic activity of the neonatal NADPH oxidase system may explain variances in NBT results (131, 133). Comparable bactericidal activity has been demonstrated between healthy neonatal and adult neutrophils toward *Staphylococcus aureus, E. coli, Serratia marcescens, Pseudomonas* species, and groups A and B streptococci (127), while neutrophils from stressed preterm and term infants demonstrated significantly decreased bactericidal activity against both Gram-positive and Gram-negative bacteria (127). Neutrophils from neonates with perinatal distress also exhibited respiratory burst suppression (134–136). Even though the respiratory burst normalized to adult cellular function in preterm infants by 2 months of age, it remained depressed in ill infants receiving intensive care (137).

The interaction between ROS and microorganisms is the foundation of CL. In healthy, term neonates, CL is normal or enhanced for group B *Streptococcus* (GBS) and opsonized zymosan (111, 138) but is generally dampened in neutrophils from healthy preterm neonates <34 weeks of GA (111, 130, 134, 139). Moreover, critically ill preterm or term infants have significantly reduced CL (140), as do neutrophils challenged with large bacterial loads (141). Although neonatal deficiencies in CL persist throughout the course of serious infection, it tends to normalize to adult levels by 2 months after birth (142).

### Neutrophil Extracellular Traps (NETs)

By extruding chromatin material loaded with antimicrobial molecules including citrullinated histones, elastase, myeloperoxidase, lactoferrin, and defensins extracellularly (143) through formation of NETs, neutrophils can entrap and kill bacteria, fungi, and protozoa (144). Neutrophils can produce NETs by two distinct pathways. The first is initiated in response to LPS, TNF-α, or IL-8 (145, 146) and requires activation of NADPH oxidase (147), ROS production (148, 149), and induction of the RIPK3–MLKL cascade (150, 151). Neonatal neutrophils from term and preterm infants fail to form NETs in this manner, even though they have the ability to generate endogenous ROS and have NADPH activity equivalent to adult cells (152). Hence, Lipp and coworkers demonstrated significantly less NET formation, with reduced NET area, from neonatal cord blood neutrophils compared to adult cells following stimulation with *N*-formylmethionine-leucyl-phenylalanine (fMLP), PMA, and LPS, with even lower numbers and NET area noted in preterm neonatal neutrophils (153). This may be due to NET-inhibitory factors (nNIF) or nHIF-related peptides, which appear to be unique to neonatal neutrophils and function as important regulators of fetal and neonatal inflammation (154).

Neonatal neutrophils, irrespective of GA at birth, produce NETs *via* the second, ROS-independent pathway after exposure to certain pathogens and following activation by the complement system (155, 156), TLR2, and/or fibronectin (150). Byrd and colleagues recently demonstrated that preterm and term infants as well as adult controls produced NETs equally well when exposed to fibronectin together with either purified β-glucan or *C. albicans* hyphae in a ROS-independent manner but did not form NETs when exposed to fibronectin or β-glucan independently (157).

## Neutrophils and the Microbiome

During parturition, the once naïve fetus passes through the vaginal canal where it is exposed to trillions of microorganisms that comprise the maternal microbiota. Neutrophil tolerance is imperative during this period to prevent the induction of pro-inflammatory reactions as the newborn is colonized with commensal microbes that harbor a variety of nucleic acids, proteins, and antigens. Once established, a symbiotic relationship is created between the host microbiota and neutrophils to ensure proper neutrophil function and numbers. Interventions that hinder the natural development of the newborn’s microbiota, such as cesarean delivery or exposure to intrapartum and/or postpartum antibiotics, may place the infant at an increased risk for late-onset sepsis, necrotizing enterocolitis, prolonged length of stay, and/or death (4, 158, 159). Recent discoveries by Deshmukh and colleagues help explain this association. Using a murine model, this group demonstrated that the pup microbiota induced IL-17 production by group 3 innate lymphoid cells in the intestine, which increased G-CSF and, hence, neutrophil production in a TLR-4- and MyD88-dependent manner (160). This interaction also increases the number of aged circulating neutrophils in adult mice, which possess enhanced α_M_B_2_ integrin and are more proficient at NET production under inflammatory conditions (161). Similar studies on aging neutrophils, however, are lacking in neonatal models.

## Neonatal Neutrophils and Immunologic *Quid Pro Quo*

After birth, cord blood neutrophils appear “primed” because they demonstrate increased concentrations of pro-inflammatory chemokines and cytokines, as well as experience prolonged survival secondary to deficient programmed apoptosis (162). Shortages of crucial cell membrane receptors, diminished intracellular signaling, and impaired cellular functioning, however, can result in neutrophil dysfunction leading to increased susceptibility to sepsis, tissue damage, and shock. Well-documented examples of enhanced neutrophil inflammatory responses with associated inhibitory mechanisms are outlined below.

Unstimulated cord blood neutrophils from term, healthy neonates have higher concentrations of pro-inflammatory cytokines such as IL-1β, TNF-α, and IFN-γ compared to adult controls, irrespective of labor exposure (91). In addition, neonatal neutrophils experience heightened IL-1β expression following stimulation by TNF-α and LPS as compared to adult cells (163). When toll-like receptors (TLRs) 1–9 are directly stimulated, however, neonatal neutrophils exhibit a global decrease in the production of these T_H_1-polarizing cytokines, which are vital in protecting the newborn against intracellular viral and bacterial infections. Instead, neonatal neutrophils secrete greater amounts of T_H_2-polarizing cytokines, including IL-6 and IL-10, which are adapted to defend against parasitic infections but can also increase the newborn’s future risk of developing atrophy, allergy, and asthma (9, 164, 165). The reasons for their decreased responsiveness to T_H_1-mediated responses may include reduced intracellular mediators of TLR signaling (166) or increased levels of plasma adenosine (9).

It has been proposed that adenosine, by binding G protein-coupled A3 adenosine receptors (A3ARs), increases intracellular cAMP levels, thereby preserving generation of T_H_2-polarizing cytokines, including IL-6, which have anti-inflammatory properties and can impede neutrophil migration to site of inflammation (9). It is known, however, that A3ARs couple to the G protein G_i_, which inhibits adenylyl cyclase and lowers cellular cAMP levels (167). Thus, it is possible that alternate G protein coupling may occur in neutrophils or that adenosine may facilitate IL-6 generation through a different member of this receptor family. Although adenosine suppresses neutrophil cell membrane levels of CD11b in neonatal and adult neutrophils, their ability to phagocytose microorganisms remains unaffected. Unlike adult cells (168, 169), however, adenosine does not alter chemotaxis or production of ROS by neonatal neutrophils (170). Nonetheless, adenosine increases susceptibility to infection by intracellular pathogens through TH2-polaring pathways while facilitating colonization of commensal microorganisms after birth by limiting excessive inflammation.

As previously noted, cell membrane levels of galectin-3 are greatly increased in neonatal compared to adult neutrophils (80). Additionally, unstimulated neonatal cells also produced larger amounts of the potent chemoattractant IL-8 (171). Following labor exposure, both galectin-3 and IL-8 concentrations are notably higher in neonatal neutrophils, leading investigators to conclude that fetal neutrophils reside in a “pre-primed” state and become reactive following labor. When stimulated with LPS, however, no differences in neutrophil galectin-3 or IL-8 levels were found, whether or not the neonate was exposed to labor. Importantly, neonatal neutrophils also did not demonstrate improved L-selectin shedding (80). Yost and colleagues also showed similar lack of upregulation of pro-inflammatory mediators after TLR 1–9 stimulation but did demonstrate elevated IL-8 levels using the TLR1/2 heterodimer agonist, PAM_3_CSK_4_ (172). While not directly tested in neutrophils, TLR 1/2 activation by PAM_3_CSK_4_ in monocytes is associated with significantly increased production of IL-10 (173), thereby blunting pro-inflammatory T_H_1 reactions in favor of T_H_2 immune responses. Thus, elevated levels of IL-8 should result in the recruitment of additional activated neutrophils to area of inflammation or infection, amplifying the acute inflammatory response with the potential to propagate local tissue damage. Without the shedding of L-selectin, however, neutrophils experience impaired rolling, firm adhesion, and endothelial transmigration, limiting their accumulation in inflamed tissue. Nonetheless, this dysregulated local response may lead to tissue damage that could result in chronic lung disease or necrotizing enterocolitis, although more research is needed to investigate this association *in vivo* (169, 174).

Neonatal neutrophils also have exaggerated pro-inflammatory responses to the major cell component of Gram-positive bacteria, peptidoglycan. Exposure to peptidoglycan stimulates neonatal neutrophil expression of CD11b, TNF-α, and IL-8, which improves chemotaxis capabilities and increases ROS production. These actions are facilitated *via* heat shock proteins, including HSPA1A and OLR1 (175). Conversely, CR3 (CD11b/CD18, MAC-1), an essential pathogen recognition receptor for Gram-negative bacteria, can bind LPS, thereby enhancing neutrophil phagocytic capabilities of these microorganisms. At baseline, however, cell membrane quantities of CR3 are decreased in neonatal cells, particularly in preterm infants, which may impair their ability to detect Gram-negative pathogens. Furthermore, once activated, neonatal neutrophil CR3 levels remain reduced compared to adult cells, which not only inhibits neutrophil activation but may also limit their accumulation at sites of inflammation (97).

Group B *Streptococcus* remains the leading cause of neonatal EOS and can elicit variable immunologic responses in the host. For example, GBS hemolysin and inflammasome components can trigger pro-IL-1β processing and IL-1β release by neonatal neutrophils, amplifying their recruitment to sites of infection (176). Alternatively, molecular mimicry by GBS capsular sialic acid and β protein attenuates innate immune responses by binding to inhibitory sialic acid-binding immunoglobulin-like lectin receptors (Siglecs) on neutrophils, triggering SHP-2 phosphatase-dependent signaling to impede neutrophil activation and phagocytic killing (177, 178). Finally, GBS can induce IL-10 production in murine pups compared to adult animals, which impairs neutrophil recruitment to inflamed tissues and reduces bacterial clearance. Attenuation of this response, however, is exhibited by TLR2-deficient murine pups, which experience improved survival by limiting GBS bacterial dissemination through enhanced GBS phagocytosis (179).

Anti-inflammatory neutrophil granular proteins, such as olfactomedin-4 located in specific granules, can also attenuate neutrophil bacterial killing and host innate immunity against Gram-negative and Gram-positive bacteria (180). Its expression is significantly upregulated in unstimulated cord blood neutrophils from healthy term newborns compared to adult cells (91) and levels are dramatically higher in septic neonates (181). OLFM-4 restricts neutrophil cathespsin c-mediated protease activity and Nod-like receptor-mediated NF-κB activation, thereby restricting antimicrobial killing (180). Elevated OLFM-4 expression is associated not only with decreased levels of IL-1β, IL-6, IL-12p40, CXCL2, G-CSF, and GM-CSF but also with greatly increased sepsis-related mortality (180, 182).

Finally, bacterial and host pro-inflammatory mediators can prolong neutrophil survival by delaying apoptosis. While this response is crucial for competent early innate immune responses that facilitate bacterial clearance, it may also promote excessive tissue injury resulting in poor neonatal outcomes (183). Allgaier and colleagues (184) have demonstrated that the overexpression of IL-1β and IL-8 cause activation of NF-κB and induction of anti-apoptotic genes. Additionally, neonatal neutrophils have diminished cellular expression of Siglec-9 and its downstream signaling protein SHP-1, an inhibitory tyrosine phosphate with proapoptotic functions (185). Inflammation resolution and tissue repair are generally facilitated by removal of toxic neutrophils through pathogen-induced programmed cell death *via* apoptosis with subsequent clearance by tissue macrophages and monocytes. The consequences of delayed neutrophil apoptosis and turnover are clearly demonstrated by Grigg and colleagues (186) who showed direct correlation with higher rates of chronic lung disease and pulmonary injury.

## Conclusion

Annually, an estimated 400,000 neonates, or 1 in 10 newborns, are born prematurely in the United States (187, 188), and prematurity-related health issues account for an astonishing 36% of all infant deaths (189). Infectious disease is the second leading cause of neonatal mortality worldwide, preceded only by complications related to preterm birth (190). Technological advancements in ventilator management, thermoregulation, nutrition, and medical therapies (such as surfactant) have permitted survival of extremely preterm infants to the limits of viability, now considered 22–23 weeks of GA or slightly beyond the halfway point of normal gestation. Nature, however, did not intend fetal survival outside the womb at this young age as evidenced by the underdevelopment of all organ systems, which increases the neonate’s risk for infection, intraventricular hemorrhage, retinopathy of prematurity, patent ductus arteriosus, necrotizing enterocolitis, chronic lung disease, and impaired neurodevelopmental outcomes. While adaptive responses are generally deficient, certain aspects of innate immunity may be absent, including the vernix that develops around 28 weeks’ GA and the stratum corneum in the third trimester (191). As with other organ systems, postnatal neutrophil deficits are exacerbated in the most immature neonates, resulting in a 10-fold greater risk for early infection compared to term infants and 30% mortality rate in those infected (191–194).

Neonatal neutrophils have been extensively studied and are often inappropriately characterized as “dysfunctional” when compared to adult cells. Because considerable differences between fetal and adult physiology exist, phenotypic and functional variances are vital. Neonatal neutrophils have adapted and evolved over time to endure extremely hypoxic *in utero* environmental conditions without triggering HIF-1α-mediated pro-inflammatory responses (195–197). Furthermore, neutrophil suppression is essential for the naïve newborn to establish a healthy microbiome in the postpartum period, but detrimental if unable to mount sufficient pro-inflammatory reactions if exposed to pathogenic organisms.

The evolution of laboratory techniques over the last 50 years has enabled researchers to substantially expand our knowledge of neutrophil biology. Neutrophils are no longer viewed as short-lived, indiscriminate phagocytes of the immune system, but instead as essential components necessary for proper B and T cell function, antigen presentation, and tissue repair and regeneration. Differences in laboratory methods and variations in neonatal populations over time may make comparisons between past and present data difficult and yield potentially contrasting results. The rapid evolution of neonatal care, from its first appearance in the 1960s to the present, may also challenge current concepts of neonatal neutrophil biology as younger, sicker babies are resuscitated, pushing the limits of viability to even lower GAs. As scientists continuously strive to discover novel therapies to enhance neutrophil function during neonatal sepsis, efforts and resources must also be dedicated to unraveling the mysteries of neutrophil biology during fetal development, taking into account environmental and compositional influences.

## Author Contributions

SL researched and composed this review. RC created the figures and edited the final version of the review. VN helped compose and edited the final version of the manuscript.

## Conflict of Interest Statement

The authors declare that the research was conducted in the absence of any commercial or financial relationships that could be construed as a potential conflict of interest.

## Abbreviations

AP, adaptor protein complex; BPI, bactericidal/permeability-increasing protein; CGD, chronic granulomatous disease; CL, chemiluminescence; EOS, early-onset sepsis; fMLP, *N*-formylmethionine-leucyl-phenylalanine; GA, gestational age; GBS, group B *Streptococcus*; GGA, Golgi-localized γ-adaptin ear homology ARF-binding protein; G-CSF, granulocyte colony-stimulating factor; GM-CSF, granulocyte-macrophage colony-stimulating factor; HIF, hypoxia-inducible factor; HSC, hematopoietic stem cell; ICAM, intercellular adhesion molecule; IVIG, intravenous immunoglobulin; LAD, leukocyte adhesion deficiency; LPS, lipopolysaccharide; LFA-1, lymphocyte function-associated antigen-1; NET, neutrophil extracellular trap; nNIF, NET-inhibitory factors; NBT, nitroblue tetrazolium; NRP, nHIF-related peptides; PMA, phorbol myristate acetate; RAGE, receptor for advanced glycation endproducts; ROS, reactive oxygen species; SGA, small for gestational age; SNARE, soluble *N*-ethylmaleimide-sensitive factor attachment protein receptor; TLR, toll-like receptor; VCAM-1, vascular cell adhesion molecule 1.
